# *Neurobiology of Language*: Reviewers List

**DOI:** 10.1162/nol_e_00064

**Published:** 2021-12-23

The editorial board of *Neurobiology of Language* appreciates the time and effort of reviewers who generously share their expertise during the peer-review process, offering substantial guidance to authors to help improve the quality of published manuscripts. Reviewer contributions are critical to the goals set forth for the journal, and we thank all who participated during the first two years of the journal’s existence. Below is a list of reviewers for 2020–2021.

F.-Xavier Alario

Phillip Alday

John Anderson

Richard Arenas

Sharon Ash

Florencia Assaneo

Ryszard Auksztulewicz

Giosuè Baggio

Thomas Bak

Valentina Bambini

Elise Barbeau

Jana Basnakova

Anne Beatty-Martinez

Pascal Belin

Iris Berent

Yanchao Bi

Gavin Bidelman

Jeffrey Binder

Ferdinand Binkofski

Esti Blanco-Elorrieta

Idan Blank

Henrike Blumenfeld

Leonardo Bonilha

Ina Bornkessel-Schlesewsky

Heather Bortfeld

Olga Boukrina

Mathieu Bourguignon

Francesca Branzi

Jonathan Brennan

Christian Brodbeck

Trevor Brothers

Bradley Buchsbaum

Laurel J. Buxbaum

Karen Campbell

Chiara Cantiani

Stefano Cappa

Jesús Cespón

Dustin Chacon

Ya-Ning Chang

Lang Chen

Jen-Kai Chen

Yiya Chen

Jennifer Chesters

Max Coltheart

David Copland

David P. Corina

H. Branch Coslett

Marc Coutanche

Claudia Cramer

Alessandro D'Ausilio

Wouter de Baene

Greig de Zubicaray

Mathieu Declerck

Katharine DeLong

Vincent DeLuca

Andrew DeMarco

Dirk Den Ouden

Anthony Dick

Bérengère Digard

Suzanne Dikker

Nai Ding

E. Susan Duncan

Giovann Egidi

Natalia Egorova

Else Eising

Karen Emmorey

Xiaoping Fang

Kara Federmeier

Evelina Fedorenko

Stephanie Forkel

Julius Fridriksson

Andrea Gajardo-Vidal

Jack Gallant

Zhiyao Gao

Gareth Gaskell

Avniel Ghuman

Lisa Goffman

Narly Golestani

Vincent Gracco

David Green

Michel Grothe

Adrian Guggisberg

Jason Gullifer

Laura Gwilliams

John Hale

Roy Hamilton

Roeland Hancock

John Hart

Gesa Hartwigsen

Denise Harvey

Olaf Hauk

Stefan Heim

Mireia Hernández

Arild Hestvik

Julia Hofweber

Judith Holler

Thomas Hope

Rachael Hulme

E. Matthew Husband

Alexander Huth

Rebecca Jackson

Victoria Jackson

Marc Joanisse

Marina Kalashnikova

Rajesh Kana

Katerina Kandylaki

Olga Kepinska

Andrew Kertesz

Aneta Kielar

Kwang Seob Kim

Denise Klein

Daniel Kleinman

Xiangzhen Kong

Genevieve Konopka

Sonja Kotz

Shanna Kousaie

Ioulia Kovelman

Jens Kreitewolf

Katya Krieger-Redwood

Saloni Krishnan

Vicky Lai

Matthew Lambon Ralph

Sarah Laszlo

Ellen Lau

Minna Lehtonen

Gari Lerma

Einat Liebenthal

Antonietta Liuzzi

Gigi Luk

Cheng Luo

Fabio Macciardi

Sarah MacPherson

Mandy Maguire

Alec Marantz

Paula Martin

William Matchin

Urs Maurer

Ludo Max

Mary McAndrews

Genevieve McArthur

Kathleen McCarthy

Lars Meyer

Kelly Michaelis

Michelle Moerel

Nicola Molinaro

Philip Monahan

Angela Morgan

Riikka Mottonen

Yun Nan

Nicole Neef

Charles A. Nelson

Andrew Nevins

Thomas Nichols

Mante Nieuwland

Caroline Niziolek

Anni Nora

Pedro Paz-Alonso

Fabian Pedregosa

Jonathan Peelle

Martin Pickering

David Plaut

Christos Pliatsikas

Stephen Politzer-Ahles

Chantel Prat

Milena Rabovsky

Jamie Reilly

Jessica Richardson

Amelie Rochet-Capellan

Jennifer Rodd

Antoni Rodriguez-Fornells

Ardi Roelofs

Daniela Sammler

Andrea Santi

Sebastian Sauppe

Gottfried Schlaug

Matthias Schlesewsky

Julie Schneider

Ben Schultz

Nuria Sebastian-Galles

Katrien Segaert

Valerie Shafer

Shereen Sharaan

Daniel Sharoh

Einat Shetreet

David Soto

Emmanuel Stamatakis

Brielle Stark

Alena Stasenko

Kristof Strijkers

Meghan Swanson

Jakub Szewczyk

Hiromasa Takemura

Jakke Tamminen

Xiangbin Teng

Cynthia Thompson

Kalinka Timmer

Francesco Tomaiuolo

Joe Toscano

Katherine Travis

Pascale Tremblay

Nathalie Tzourio-Mazoyer

Kenneth Vaden

Janet van Hell

Rik Vandenberghe

Kelly Vaughn

Pablo Vidal

David Vinson

Toms Voits

Matthew Walenski

Bridget Walsh

Carol Wang

Jason Warren

Kate Watkins

Sandra Weintraub

Janet Werker

Roel Willems

Stephen Wilson

Charlie Wiltshire

Patrick Wong

Malte Wöstmann

Ming Xiang

Essa Yacoub

Huichao Yang

Linmin Zhang

Benedikt Zoefel

